# Micro-Organisms and Cell Action in the Animal and Vegetable World—Pathologically Considered

**Published:** 1894-02

**Authors:** A. Bethune Patterson

**Affiliations:** 204 Equitable Building; Atlanta, Ga.


### MICRO-ORGANISMS AND CELL ACTION IN THE ANIMAL AND VEGETABLE WORLD—PATHOLOGICALLY CONSIDERED.

BY A. BETHUNE PATTERSON, M. D., ATLANTA, GA.

Medicine becomes a science when the phenomena, observed and classified as symptoms, are traced to the operation of laws which, when analyzed, are shown to be fixed and constant, and which investigations demonstrate are inseparable from pathological conditions. The study of these laws, and their manifestations, has had the effect of revolutionizing medicine. By the modern methods of observation and study, surgery has taken great strides, and is now firmly planted upon a scientific basis. To this progress is due the new-born branch, bacteriology. It is in this field that the biologist reaps a rich harvest, and physiology is given new life. There are few forces in nature, and the laws governing, that are clearly understood, either in the animal, vegetable, geological or mineral world. In investigating physiological, etiological and pathological conditions, we have confronting us those mysterious influences which come under the head of biological law. In the animate world we observe laws operating, when environments favor, producing manifestations of certain phenomena called life. In the animal and vegetable kingdom we speak of the reproduction of cells as an act of germination. It is with this law that we have to deal in studying not only physiological phenomena, but the etiological factors in pathological conditions; so it may be understood that biology bears with equal force upon physiological and pathological phenomena, and the law of life becomes the law of disease and death. In analyzing these conditions we are unavoidably led into nature's labyrinth of action; the cells, the elements of these bodies (protoplasm, etc), are observed to be in constant motion and activity, and in many of them, if not all, the power of reproduction is inherent, the intricate and various functions of organs are herein elaborated, and the interruption of the normal action of cells is always observed in diseased processes. Microscopical pathology or morbid anatomy has led to the study of micro-organisms, which are generally classified as bacteria. Many of these do not differ, either in structure or methods of reproduction, from cells which go to make up the tissues and organs of the body. A little investigation along this line would remove any doubt that might exist in the minds of students of medicine as to the relation micro-organisms bear to disease. The objections raised by skeptics to the germ theory of disease prove how unfamiliar they are with the subject. Many of the older members of the profession are averse to entering this field of study; the mere suggestion excites their ridicule. This class of bigots are in a position to add little, if anything, to scientific medicine, unless it be that form of stimulus so necessary in every field, derived from opposition. It is to be deplored when such men are found occupying the position of instructor in institutions of learning, where the groundwork of a progressive branch of science is being laid. Youth overflows with veneration for the furrowed face and hoary locks, and fosters impressions from such a source, which often prove lasting. The cause of disease becomes more intelligible with the daily progress of bacteriological research, and in consequence much of the empiricism which has taken an active part in the management and treatment of disease is giving place to more rational and scientific methods. A well-known medical writer and lecturer attempts to explain the action of a certain drug in overcoming and eliminating a specific form of disease. He rules out the germ theory of disease and prefers to deal with the cause as an "unknown quantity" and claims that it progressively multiplies. A reflection upon the law governing the phenomena of life would be conclusive that this unknown quantity could not be classified with the inanimate, but the animate world, and must be found to stand in the animal or vegetable series, be it ptomaine or leucomaine. A little thought would relieve the student of embarrassment, as without the micro-organism and its germination there could not be ptomaines or leucomaines; if shown that either of the latter possess the toxical effect disturbing the equilibrium of physiological processes, the origin of such agents, being in itself harmless, would not render the microbe less the object of interest. The inability, in certain forms of disease, to isolate, or cultivate the specific form of bacteria cannot be utilized as an argument against the existence of such cases, because the phenomena of germination being present is proof of the existence of life. Pathological bacteria have been isolated, and in an unadulterated condition transplanted into the tissues of animals, proving conclusively that the germination following is inseparable from the pathological changes observed to take place.

It is well known that certain micro-organisms attack the living cells, inhabit their bodies and feed upon the nutritious elements; a dissolution of cells follows, as an example, the malarial germs attack the red corpuscles of the blood; the examination of the blood in ague patients shows that there is a destruction of the red disks and the setting free the hæmoglobin. Medical writers for ages resorted to the method of classifying diseases. This grew out of the observed similarity, both in the objective and subjective expressions of the disease processes. In the animal and vegetable kingdom of large dimensions—so in the microscopical world we observe genus. With the bacteriologist this classification of species is as harmonious as that formulated by the botanist and naturalist.

Before proceeding further I wish to say, that I am constrained to look to the spiritual forces pervading all nature as explanatory of the unknown activity of the cells, laws that are receiving but little attention from the scientist. Investigations show that micro-organisms of a low form of life do not differ in their structure from animal and vegetable cells. Take the moulds or hyphomicete, yeast or blastomicetes, the schizomycetes or fission fungi; here we have several forms of life capable of reproduction, though by different methods. For instance, the schizomycetes are single cells, reproducing by division or fission in some of the simpler forms of moulds; conidia are formed directly from the hyhæ of the mycelium. In yeast the cells multiply by budding; the new cells seem to grow out of the parent cell, and drop off or form a chain of cells. The same law holds in bacteria. In some the reproduction is by division, in others by spore-formation; spores form in the interior, in other species the cells seem to split up into spores. In considering these forms of life from a pathological standpoint, it is well to note the difference between the mother cell and the spores, a difference of harmlessness. A similar difference exists between an egg and the parent bird. When spores or germs are properly placed they quickly develop into active cells or micro-organisms. The acarus scabiei, one-twenty-fifth of an inch in length, may be taken as one of the largest forms of parasitic life, which produce very annoying pathological conditions. The female penetrates into the epidermis and deposits her eggs, which quickly germinate; the itching, scratching and the characteristic lesion immediately follow. A step down the scale, we meet with moulds, pathogenic, and of these we have trichophyton tonsurans, familiarly known as ringworm, tinea circinata of the skin, tinea sycosis of the beard. Saccharomyces albicans are oval, round cells of the yeast variety, and multiply by budding. Pathologists have ascribed to these cells the peculiar and familiar pathological condition found in the mouth of infants, commonly known as thrush. The schizomycetes are an interesting class of micro-organisms, inasmuch as they are found in great abundance everywhere. In fact, it might be said their absence in any medium is evidence of sterilization. We are led to conclude that animal and vegetable life is dependent upon animal and vegetable elements of nutrition for sustenance, and without them the species would become extinct; on the other hand their existence is jeopardized by similar forms of life. This antagonism can be traced from the very largest and most complex forms of life down to the smallest microscopical beings. Diseased conditions must be recognized to be the result of this form of antagonism.

204 *Equitable Building.*

